# Revisiting the Sequence Method for Baroreflex Analysis

**DOI:** 10.3389/fnins.2019.00017

**Published:** 2019-01-23

**Authors:** Luiz Eduardo Virgilio Silva, Daniel Penteado Martins Dias, Carlos Alberto Aguiar da Silva, Hélio Cesar Salgado, Rubens Fazan

**Affiliations:** ^1^Department of Physiology, Ribeirão Preto Medical School, University of São Paulo, Ribeirão Preto, Brazil; ^2^Barão de Mauá University Center, Ribeirão Preto, Brazil

**Keywords:** heart rate variability, autonomic nervous system, baroreflex, sequence method, sensitivity, effectiveness index

## Abstract

The sequence method is an important approach to assess the baroreflex function, mainly because it is based on the spontaneous fluctuations of beat-by-beat arterial pressure (for example, systolic arterial pressure or SAP) and pulse interval (PI). However, some studies revealed that the baroreflex effectiveness index (BEI), calculated through the sequence method, shows an intriguing oscillatory pattern as function of the delay between SAP and PI. It has been hypothesized that this pattern is related to the respiratory influence on SAP and/or PI variability, limiting the SAP ramps to 3 or 4 beats of length. In this study, this hypothesis was tested by assessing the sequence method using raw (original) and filtered series. Results were contrasted to the well-established transfer function, estimated between SAP and PI. Continuous arterial pressure recordings were obtained from healthy rats (*N* = 61) and beat-by-beat series of SAP and PI were generated. Low-pass (LP) and high-pass (HP) filtered series of SAP and PI were created by filtering the original series with a cutoff frequency of 0.8 Hz. Original series were analyzed by either the sequence method or cross-spectral analysis (transfer function) at low- (LF) and high- (HF) frequency bands, while filtered series were evaluated only by the sequence method. Baroreflex sensitivity (BRS) and BEI of original series, calculated by sequence method, was highly (85–90%) determined by HP series, with no significant association between original and LP series. A high correlation (>0.7) was found between the BRS estimated from original series (sequence method) and HF band (transfer function), as well as for LP series (sequence method) and LF band (transfer function). These findings confirmed the hypothesis that the sequence method quantifies only the high-frequency components of the baroreflex, neglecting the low-frequency influences, such as the Mayer waves. Therefore, we propose using both the original and LP filtered time series for a broader assessment of the baroreflex function using the sequence method.

## Introduction

The sequence method, first described in the mid-1980s, was a milestone for the analysis of baroreflex function at both clinical and experimental levels (Bertinieri et al., 1985). First, because it relies on the spontaneous fluctuations of beat-by-beat arterial pressure (AP) and cardiac interval, i.e., this approach does not require the induction of blood pressure changes, avoiding many drawbacks related to it. Second, the sequence method not only evaluates the baroreflex sensitivity (BRS) but also provides the baroreflex effectiveness index (BEI). In contrast to BRS, BEI reflects the percentage of beat-by-beat AP changes that are effectively translated into reflex changes of the heart rate (HR; Di Rienzo et al., 2001). Therefore, the BEI is considered a complementary index to the BRS, providing additional information regarding baroreflex function (Lataro et al., 2017; Silva and Katayama, 2017).

In the late 2000s, Laude and coworkers studied more in-depth the parameters of the sequence method to better understand their influence and the best choices for working with mice (Laude et al., 2008, 2009). They also reported an intriguing feature: when the BEI is calculated for increasing delays between beat-by-beat systolic AP (SAP) and pulse interval (PI), its value oscillates with a period of 3 to 4 beats. On the other hand, the BRS, also assessed with increasing delays, do not show any oscillatory pattern.

**FIGURE 1:**
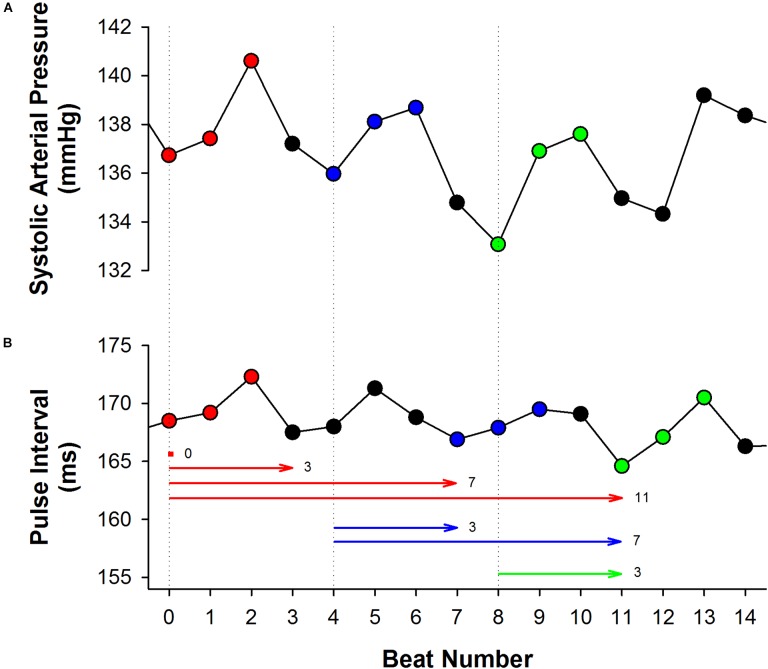
The effect of respiration on the sequence method. In both excerpts of systolic arterial pressure (SAP, **A**) and pulse interval (PI, **B**) time series, obtained from a healthy rat, it is noticeable the influence of respiration, increasing and decreasing beat-by-beat SAP and PI with a period of three to four beats. Three up ramps were found in the SAP series, as shown in red (first ramp), blue (second ramp) and green (third ramp). The first up PI ramp found after each up SAP ramp was highlighted using the same color. Nevertheless, the sequence method may associate several PI ramps to a given SAP ramp, depending on the delay adopted (horizontal lines). For instance, the first up SAP ramp (red) can be associated with the PI ramps found in delays 0, 3, 7, and 11. Similarly, the second SAP ramp (blue) can be associated with the PI ramps found after 3 and 7 beats of delay. The same behavior occurs with the third SAP ramp. Therefore, BEI is expected to be higher for delays 0, 3, 7, and 11. In other words, BEI will oscillate with a period of three to four beats, due to the repetitive nature of the ramps imposed by the respiration.

Even though these studies did not address the underlying causes of this oscillatory profile of BEI for incremental delays, we hypothesized that it might be a consequence of the respiratory influence on SAP, as highlighted in a recent study of our group (Lataro et al., 2017). The respiratory cycle in rats and mice lasts about 4 to 5 beats and the respiratory driven changes in SAP can often be easily identified (Figure 1). As consequence, SAP ramps (up or down) are usually limited to the length of 4 or 5 beats. In other words, a delay of 3 to 4 cardiac intervals is expected to separate SAP ramps. Since SAP ramps drive baroreflex-mediated changes in HR, one can expect the same 3 to 4 cardiac intervals separating one PI ramp to another. Although each SAP ramp is believed to elicit only one ramp of PI (the reflex response), the sequence method may erroneously associate many PI ramps at a given SAP ramp, leading, for instance, to oscillation of the BEI values every 3 or 4 PIs (see Figure 1).

Therefore, it seems that the sequence method is limited to assess spontaneous baroreflex function only at AP oscillations modulated by respiration, which limits the size of the SAP ramps. This concept carries important consequences, as the sequence method ignores the baroreflex responses to slow AP changes, such as the Mayer waves (Penaz, 1978). The current study evaluated the influence of low- and high-frequency oscillations of SAP and PI in the sequence method, and compared the BRS calculated using the sequence method to BRS obtained from the cross-spectral analysis. Also, we proposed an alternative approach to use the sequence method to calculate both the fast and slow components of the baroreflex.

## Materials and Methods

### Animals

The dataset of this study is composed by revisited AP recordings (*N* = 61) from normal male young adult Wistar rats (250–300 g), obtained from previously published (Dias et al., 2016; Silva et al., 2017) and unpublished studies. In all experiments, the animals were maintained under controlled light (12–12 h light-dark cycle) and temperature (21°C) environment with water and food provided *ad libitum*. All experimental procedures adhered to the Guide for the Care and Use of Laboratory Animals prepared by the National Academy of Sciences and published by the National Institutes of Health and were approved by the Committee of Ethics in Animal Research from the Ribeirão Preto Medical School – University of São Paulo, Ribeirão Preto, SP, Brazil.

### Experimental Procedures

Rats were anesthetized with a mixture of ketamine (50 mg/kg) and xylazine (10 mg/kg) and instrumented with a polyethylene catheter inserted into the carotid artery (*N* = 7), femoral artery (*N* = 43) or aorta (*N* = 11) for continuous direct AP recordings. Some rats also received a catheter inserted into the femoral vein (Silva et al., 2017) or subcutaneous electrodes for ECG recordings (Dias et al., 2016), according to the needs of the experimental protocol. 24 to 48 h after the surgical procedures, the arterial line of the animals was connected to a pressure transducer (MLT844, ADInstruments, Bella Vista, NSW, Australia) attached to a Bridge Amp (FE221, ADIstruments, Bella Vista, NSW, Australia) and the AP was continuously sampled (2 kHz) for 30 min, in an IBM/PC through an analogic to digital interface (Power Lab 4/40, ADInstruments, Bella Vista, NSW, Australia). All the recordings were performed in unanesthetized freely moving animals kept in individual cages and during basal conditions.

### Data Pre-processing

Approximately 20 min of stable AP recordings from each rat was processed by computer software (LabChart Pro, ADInstruments, Bella Vista, NSW, Australia) to generate beat-by-beat series of SAP and PI values. Artifacts were removed from each series using the following procedure: a moving median window of 50 points was used to calculate the series *baseline*. Upper and lower thresholds were set as: *baseline* ±*p* ∗*baseline* (0.05 < *p <* 0.20). All values that exceeded the thresholds (upper or lower) were removed from the series. Removals never exceeded 1% of the original time series length. The average ± SD of series length is 8.240 ± 3.590 beats.

### Low- and High-Pass Filtered Series

**FIGURE 2:**
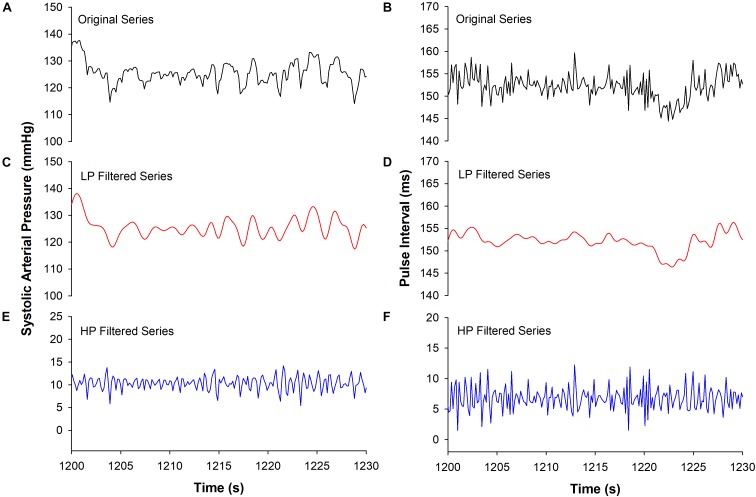
Example of original and filtered series. Excerpts of SAP are shown for original **(A)**, low- (LP, **C**) and high-pass filtered (HP, **E**) series, as well as excerpts of PI for original **(B)**, low- **(D)** and high-pass filtered **(F)** series.

Filtered versions of the SAP and PI series were created by filtering the original series using a 9th order Butterworth filter with a cutoff frequency of 0.8 Hz. Low-pass (LP) and high-pass (HP) filtered series were created in order to determine the influence of low and high-frequency components of the baroreflex in the sequence method. The cutoff frequency was chosen according to the spectral components of the rat cardiovascular variability (Cerutti et al., 1991, 1994). Figure 2 shows examples of the original and filtered SAP and PI series.

### Sequence Method

The sequence method assumes that successive spontaneous increases or decreases in beat-by-beat AP values (here, SAP ramps) elicit baroreflex-mediated responses in the PI length (Laude et al., 2008). Therefore, to evaluate baroreflex function, this approach searches for SAP ramps linked (linearly correlated) to changes in PI. Some parameters need to be set in order to apply the sequence method. First, a minimum variation (threshold) for SAP or PI change needs to be determined, i.e., the differences between the successive values of SAP or PI must meet a defined threshold. Second, the minimum sequence length (*n*), and the delay between SAP and PI ramps (*d*) must also be chosen. Finally, a minimum correlation coefficient (*r*) between SAP and PI ramps must be achieved to consider an actual baroreflex sequence. In other words, when an SAP ramp of *n* consecutive values (up or down) correlates with PI changes at the same direction, delayed by *d* beats from the SAP ramp, a baroreflex sequence has been found. The BRS (or gain) of each sequence is calculated by the slope of the regression line of PI vs. SAP, and the gain of the animal is the average slope, calculated from all the sequences found.

Besides the BRS, the sequence method also provides an additional index of baroreflex function, namely the BEI (Di Rienzo et al., 2001). BEI is the ratio of the number of sequences and the number of SAP ramps, occurring between 0 and 1. In brief, BEI depicts how many of the SAP changes are effectively translated into a change in PI, independently of its magnitude. Therefore, BEI and gain (i.e., slope) provide markers on different aspects of the spontaneous baroreflex function.

Following the guideline from a previous study (Laude et al., 2008, 2009), we set the correlation threshold to *r* = 0.8 and the thresholds for SAP or PI changes were set to zero (i.e., any change in SAP or PI is considered). The delay between SAP and PI was assessed from 0 to 12 beats. The minimum sequence length was set to *n* = 3 for original time series and varied from *n* = 3 to *n* = 9 for filtered series. BRS and BEI were calculated using the computer software CardioSeries v2.4^1^ (Dias et al., 2016).

### Cross-Spectral Analysis by Transfer Function Estimation

The transfer function is a mathematical estimation that represents how a given system responds (output) to inputs (Pinna and Maestri, 2001). The baroreflex system is sensitive to AP changes (input), responding with changes in cardiac interval length. Therefore, estimating the transfer function between SAP and PI series provides information about the baroreflex function (Porta et al., 2013a).

The transfer function, which is defined in the frequency domain, can be estimated by the ratio of the PI-SAP cross-spectrum to SAP spectrum, and its modulus represents the gain of the baroreflex (Porta et al., 2013a). In addition to the transfer function, the coherence between SAP and PI can also be calculated to identify those frequencies where SAP and PI are more “coherent” or coupled. Therefore, under frequencies where those two signals are not coupled, the transfer function may be disregarded (Pinna and Maestri, 2001, 2002).

The original SAP and PI series were interpolated at 10 Hz (cubic spline) to become evenly spaced in time and were divided into half-overlapping segments of 4096 data points. This procedure is the well-known Welch protocol (Welch, 1967). A Hanning window was used to attenuate the spectral leakage in the side-lobes of the spectra, and the spectrum of each segment was calculated using the fast Fourier transform (FFT). The transfer function (gain or BRS) between SAP and PI was integrated into low (LF: 0.2–0.8 Hz) and high frequency (HF: 0.8–3.0 Hz) bands, accounting only for the frequencies where the coherence function was greater than 0.5. While the LF band carries relevant information about Mayer’s waves of SAP, HF band mainly accounts for the respiratory oscillations of SAP (Julien, 2008). The transfer function was not estimated for the filtered time series.

### Statistical Analysis

The normality of data distribution was verified by the Shapiro–Wilk test. Multiple linear regression was used to identify which oscillatory components of the original SAP and PI series are the most relevant to the sequence method. For this purpose, the BRS (gain) of original series was modeled as a linear combination of the gain obtained from LP and HP filtered series, individually or combined. The same procedure was applied to BEI, i.e., it was modeled as a linear combination of the BEI estimated from LP and HP filtered series. The coefficient expressing how well each model describe the data (*R*^2^) was reported. In addition, Bland–Altman plots were used to illustrate the agreement between the BRS, calculated using the sequence method, obtained from original and filtered series. The Spearman correlation coefficient was estimated between the sequence method BRS and the transfer function BRS (in LF and HF bands). The Wilcoxon Rank Sum Test and Friedman ANOVA on ranks were applied to check for differences between the BRS in LF and HF band (transfer function) and the BRS of original and filtered time series (sequence method). Significant differences were assumed when *P* < 0.05.

## Results

**FIGURE 3:**
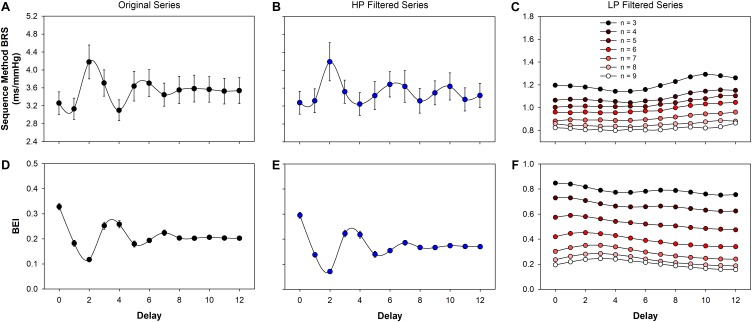
Baroreflex function estimated using the sequence method. The baroreflex sensitivity (BRS) for original **(A)**, high- (HP, **B**) and low-pass (LP, **C**) filtered is shown as function of the delay between SAP and PI. The baroreflex effectiveness index (BEI) for original **(D)**, high- **(E)** and low-pass **(F)** filtered series are also shown as a function of the delay. The oscillatory profile of BEI observed in original and HP filtered series is a consequence of the restriction imposed by respiration on the sequence length (see Figure 1). For LP filtered series, both the BRS and the BEI monotonically decreases as *n* increases. The minimum sequence length was set as *n* = 3 for original and HP filtered series and varied from *n* = 3 to *n* = 9 for LP series. The delay was assessed from *d* = 0 to *d* = 12. Values are mean ± standard error.

Both BRS and BEI, calculated using the sequence method, from the original (raw) and filtered series, are shown in Figure 3. As expected, BEI presented an oscillatory profile with a period of 3 to 4 beats for original (Figure 3D) and also for HP filtered series (Figure 3E). In the present study, BRS also showed periodic oscillation for increasing delays (Figures 3A,B). In contrast, for LP filtered series there is no clear oscillatory profile for either BEI or BRS (Figures 3C,F). However, the minimum sequence length markedly alters the BRS and BEI of LP filtered series. For *n* = 3 to *n* = 9, BRS changed from 1.2 to 0.8 ms/mmHg (Figure 3C), whereas the BEI varied from 0.8 to 0.2 (Figure 3F).

### The Sequence Method Detects Only High-Frequency Oscillations

The BRS and BEI of the sequence method, calculated from original SAP and PI series, were modeled as a linear combination of the same index calculated from LP and HP filtered series. The squared multiple correlation index (*R*^2^), representing the “quality” of each model, is reported in Table 1. Results show that the BRS (or BEI) of original series can be highly determined (explained) by the BRS (or BEI) of HP filtered series. When the BRS (or BEI) of LP filtered series is introduced into the model, no improvement is obtained. For the most used delays (*d* = 1 and *d* = 3), 85 to 90% of original BRS and BEI can be explained by the HP series, whereas only 5% could be attributed to LP filtered series. Even so, the association between original and LP filtered series is not significant for both the BRS and BEI.

**Table 1:** Multiple linear regression of the BRS (gain) and BEI estimated by the sequence method for original SAP and PI series.

	LP filtered series only		HP filtered series only		LP + HP filtered series	
			
	BRS	BEI	BRS	BEI	BRS	BEI
***d***	***R*^2^**	***R*^2^**	***R*^2^**	***R*^2^**	***R*^2^**	***R*^2^**

0	0.06	0.06	0.89^∗^	0.84^∗^	0.89^∗^	0.85^∗#^
1	0.06	0.05	0.86^∗^	0.84^∗^	0.86^∗^	0.84^∗^
2	0.01	0.01	0.62^∗^	0.80^∗^	0.63^∗^	0.80^∗^
3	0.04	0.05	0.89^∗^	0.86^∗^	0.89^∗^	0.86^∗^
4	0.08^#^	0.00	0.76^∗^	0.90^∗^	0.76^∗^	0.90^∗^
5	0.03	0.16^#^	0.72^∗^	0.91^∗^	0.72^∗^	0.91^∗^
6	0.02	0.01	0.85^∗^	0.80^∗^	0.86^∗^	0.80^∗^
7	0.05	0.01	0.66^∗^	0.81^∗^	0.66^∗^	0.82^∗^
8	0.02	0.02	0.78^∗^	0.82^∗^	0.78^∗^	0.82^∗^
9	0.04	0.02	0.91^∗^	0.81^∗^	0.91^∗^	0.81^∗^
10	0.04	0.00	0.85^∗^	0.83^∗^	0.86^∗^	0.83^∗^
11	0.03	0.09^#^	0.84^∗^	0.83^∗^	0.84^∗^	0.83^∗^
12	0.04	0.15^#^	0.85^∗^	0.67^∗^	0.85^∗^	0.69^∗^


*The gain estimated from low-pass (LP) and high-pass (HP) filtered series were taken individually or combined as independent variables of the model. The same procedure was applied, separately, for the BEI. The minimum sequence length was set to *n* = 3. BRS: baroreflex sensitivity; BEI: baroreflex effectiveness index; SAP: systolic arterial pressure; PI: pulse interval; *d*: delay of the sequence method; *R*^2^: the coefficient (between 0 and 1) expressing how well each model describe the dependent variable; ^#^*P* < 0.05 for the association between the original (dependent) and LP series; ^∗^*P* < 0.05 for the association between the original (dependent) and HP series.*

**FIGURE 4:**
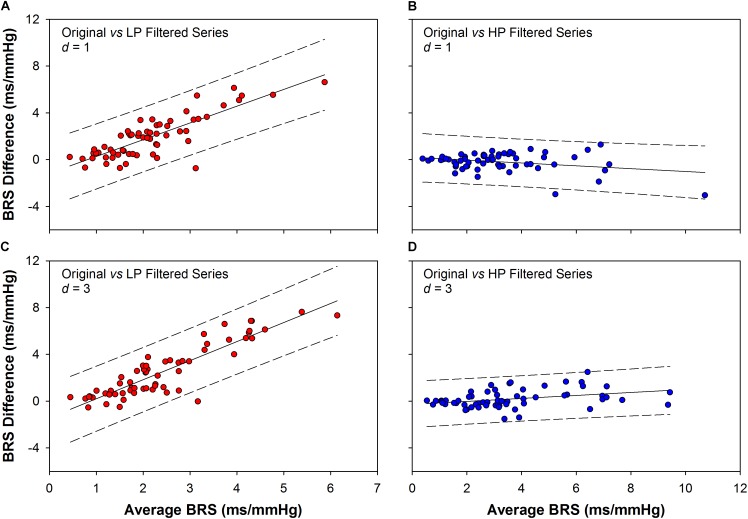
Bland–Altman plots showing the agreement between the BRS obtained from original and filtered series. The BRS difference obtained between original and low-pass (LP) filtered series is higher than the BRS difference obtained between original and high-pass (HP) filtered ones, for both *d* = 1 (1.95 ± 1.80 vs. –0.19 ± 0.79) and *d* = 3 (2.54 ± 2.24 vs. 0.19 ± 0.77, mean difference ± SD). Moreover, there is a clear proportional bias in the BRS differences between original and LP filtered series, for both *d* = 1 **(A)** and *d* = 3 **(C)**. In contrast, there is no evidence of proportional bias for the BRS difference between original and HP filtered series, as the differences show opposite and small tendencies for *d* = 1 **(B)** or *d* = 3 **(D)**. *d*: delay between SAP and PI series.

The Bland–Altman plots of the sequence method BRS are shown in Figure 4. The plots represent the agreement (interchangeability of measurements) between the BRS obtained from the original series and the BRS obtained from filtered ones. The comparison was performed for original vs. LP (Figure 4A,C) and original vs. HP filtered series (Figures 4B,D), for delays of one and three beats (*d* = 1 and *d* = 3). The agreement between the BRS of original and HP series (–0.19 ± 0.79 for *d* = 1; 0.19 ± 0.77 for *d* = 3; mean difference ± SD) is significantly higher than the agreement between the BRS of original and LP series (1.95 ± 1.80 for *d* = 1; 2.54 ± 2.24 for *d* = 3; mean difference ± SD). Moreover, there is a clear higher proportional bias for the plots between original and LP filtered BRS, indicating that the higher the BRS, the higher the difference between the two measurements.

### Correlation Between the Sequence Method and the Transfer Function

**FIGURE 5:**
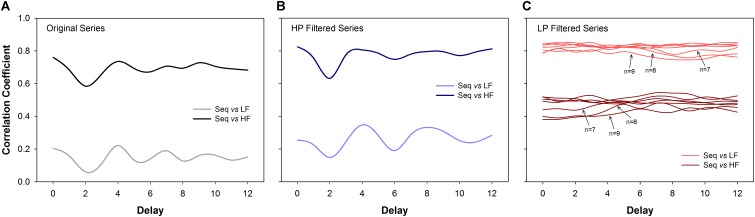
Correlation analysis (Spearman coefficient) between the sequence method and the transfer function estimation (cross-spectral analysis). For original **(A)** and high-pass (HP) filtered series **(B)**, the BRS calculated using the sequence method is much more correlated to the HF component of BRS calculated through the transfer function. For low-pass (LP) filtered series **(C)** the BRS calculated by the sequence method is more correlated to the LF component of the BRS obtained by the transfer function. The results are consistent for all delays. For LP filtered series, the correlation values tend to decrease as *n* increases. The transfer function was always estimated using the original series. LF: low-frequency; HF: high-frequency; *n*: minimum sequence length; Seq: sequence method.

The correlation coefficients between BRS calculated using the sequence method (original and filtered series) and the BRS calculated using the transfer function (LF and HF bands) are shown in Figure 5. For original (Figure 5A) and HP series (Figure 5B), the BRS calculated by the sequence method was highly correlated to the BRS estimated by the transfer function at the HF band. In contrast, the correlation between the BRS estimated from the sequence method and the transfer function in the LF band was very low. For example, for delays equal to *d* = 1 or *d* = 3 (most common choices), the correlation between BRS obtained by the two methods was in the range 0.65 to 0.78 at HF band and 0.12 to 0.24 at LF band.

The opposite was found for LP filtered time series (Figure 5C). In this scenario, BRS examined by the sequence method was strongly correlated with the transfer function at the LF band. These results were consistent for all delays and sequence length (*n*), even though the correlation showed a tendency to be lower for longer sequences.

**FIGURE 6:**
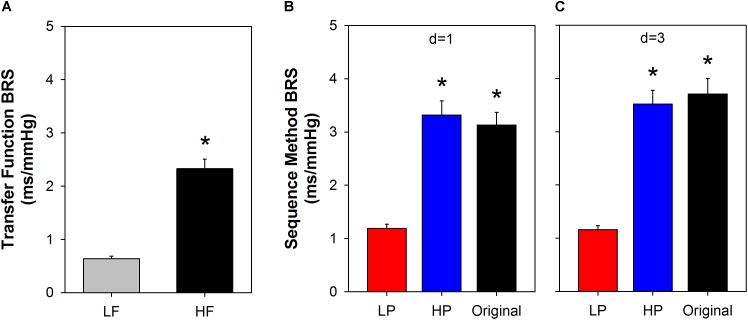
Comparison between slow and fast BRS. The BRS calculated using the transfer function **(A)** indicates that the fast components of SAP oscillations (HF band) induce a BRS that is more than three times higher the sensitivity of the slow components of SAP (LF band). Very similar results were found with the sequence method when the original or high-pass (HP) filtered series were used to measure the fast BRS components and low-pass (LP) filtered series to measure the slow BRS **(B,C)**. The minimum sequence length was set to *n* = 3 for both original and filtered series. SAP: systolic arterial pressure; LF: low-frequency; HF: high-frequency; *d*: delay between arterial pressure and PI; ^∗^ *P* < 0.05 compared to LF **(A)** or LP **(B,C)**. Values are mean ± standard error.

Figure 6 shows BRS calculated using the transfer function, in LF and HF bands (Figure 6A). For comparison, the BRS calculated by the sequence method is shown for some specific parameters (Figures 6B,C). We chose the minimum sequence length of *n* = 3 and delays of *d* = 1 or *d* = 3 beats for both original and filtered series. The mean BRS obtained by cross-spectral analysis at HF is more than three times higher compared to the mean BRS obtained at LF band (Figure 6A). The same behavior repeats for BRS calculated from original and filtered time series, using the sequence method, i.e., the BRS from original and HP filtered series are near three times higher than the BRS of LP filtered series, for both delays (Figure 6B, *d* = 1; Figure 6C, *d* = 3).

## Discussion

The analysis of spontaneous baroreflex has opened inestimable possibilities for better understanding the baroreflex function in a variety of situations. Although several methods have been designed for this purpose in the last years, the sequence method is still successfully applied and widely used (Laude et al., 2004; De Maria et al., 2018).

Nevertheless, the rupture of SAP ramps, as a consequence of the high-frequency (respiratory) fluctuations, seems to configure an important limitation of the sequence method. In other words, the method seems not to be capable of accounting for the slow components of the baroreflex. Some authors have previously reported the inability of the sequence method to account for the influence of the sympathetic system in the baroreflex (Oosting et al., 1997; Stauss et al., 2006). Corroborating this hypothesis, the multiple linear regression and Bland–Altman plots employed here showed that the BRS and BEI, estimated from the sequence method, are strictly linked to the high-frequency components of the original series. In addition, the correlation analysis showed that the BRS calculated through the sequence method (original series) is highly related to the BRS derived from the cross-spectral analysis at high – but not low – frequency range. Considering the slower nature of adrenergic transmission, preventing the sympathetic influence in AP and HR at HF band (Stauss et al., 1997; Levick, 2000), it is reasonable to say that the sequence method does not measure the sympathetic-modulated component of the baroreflex.

A reasonable alternative to measuring the slow components of the baroreflex using the sequence method is to filter out the high-frequency oscillations of SAP and PI time series (creating the LP filtered series) before applying the sequence method. In this case, the respiratory components will be removed, preventing SAP ramps to be broken every three or four beats. All analysis using LP filtered time series showed a very different scenario in comparison to the original and HP filtered ones. In one hand, the gain and BEI of HP filtered series (where only HF oscillations are present) is capable to describe, by itself, the BRS and BEI of original series; there is no significant association or agreement between the BRS of original and LP filtered series. On the other hand, the BRS of LP filtered series calculated with the sequence method is strongly correlated with the BRS at the low-frequency band of the cross-spectral analysis. Therefore, in order to measure both slow and fast components of the baroreflex utilizing the sequence method, it is conceivable to use not only the original but also the LP filtered series of SAP and PI. While results from the original series reflect the fast components of the baroreflex (the same obtained with HP filtered series), LP series will represent the slow components of the baroreflex. Of note, the cutoff frequency of filtered time series must be chosen according to the data under consideration, once the limits of LF and HF frequency bands may change for different species (Behar et al., 2018).

The best parameter choices for the sequence method was the subject of previous studies. Overall, a minimum sequence length of 3 cardiac intervals and delays of 1 or 3 beats are recommended (Laude et al., 2008, 2009). Those studies can be used as a guide for choosing the best parameters when the original time series are considered. For LP filtered time series, however, there was no guide for selecting the best values for the sequence length and delay. Moreover, the correlation between the transfer function (LF band) and sequence method (LP filtered series) was very similar for all parameters, giving no clues for their optimal values. Thus, for illustrating our proposal of using the sequence method for measuring both slow and fast components of the baroreflex, for LP filtered time series we selected the same parameters recommended for the original series, i.e., *n* = 3 and *d* = 1 or *d* = 3. However, a more comprehensive study on the effect of those two parameters must be carried out, using data obtained at diverse physiological conditions where the slow and fast baroreflex components can be controlled. For the data used here (healthy rats), only the minimum sequence length seems to play a role in the BRS and BEI of LP filtered time series. For example, the BEI showed a quite broad range of values, varying from 0.8 (*n* = 3) to 0.2 (*n* = 9) (Figure 3F). The decrease of BEI for increasing *n* is expected because the longer the SAP ramp, the lower the probability of finding a corresponding PI ramp. On the other hand, the decrease of BRS with *n* tells that shorter sequences give, in general, higher BRS than longer sequences. This is in agreement with the BRS calculated in LF and HF bands of the transfer function (higher BRS in HF band) and the overall higher BRS found for original and HP than LP filtered series, estimated by the sequence method (see Figure 6). Future studies are, therefore, necessary to identify which *n* is the best choice for the LP series, so that one can reliably quantify the BEI of the slow components of the baroreflex.

The sequence method was validated in situations of autonomic receptors blockade and by correlating the method to the classical pharmacological approach (Oxford method) (Oosting et al., 1997; Laude et al., 2004; Waki et al., 2006). However, such analyzes are not able to establish the causality between cardiovascular variables, especially when other important variables are not considered in the genesis of rhythms. For example, even though the high-frequency fluctuations of SAP and PI are highly correlated and coherent, they might be both driven by a third factor (respiration) instead of one modulating the another (Mancia et al., 1999; Faes et al., 2011). While several recent studies with causal approaches have confirmed the existence of a causal relation between SAP and PI, they also reported the existence of a causal relation from PI to SAP, as well as from respiration to SAP and respiration to PI (Faes et al., 2011; Porta et al., 2013b; Helen Mary et al., 2018). Therefore, we cannot disregard the possibility that the sequence method, calculated over original or HP series, is overestimating the BRS by not disregarding the direct and parallel influence of the respiration into SAP and PI. On the other hand, a marked reduction of the number of sequences is observed when the baroreflex is surgically removed (Bertinieri et al., 1988; Di Rienzo et al., 2001). Thus, the sequence method is able to measure the baroreflex-mediated changes from SAP to PI at high-frequencies, indeed. However, the bias of this estimation due to the influence of other factors should be investigated in further studies. In this scenario, the use of LP filtered series may configure an advantage over HP filtered ones to evaluate the baroreflex function through the sequence method, as suggested by some authors (Fazan et al., 2005; Faes et al., 2006).

### Extending the Findings to Humans

The evaluation of baroreflex function is a valuable tool in prognostic assessment and treatment strategies in a variety of cardiac diseases. However, most of the approaches developed to study baroreflex are not free of risk, requiring, for example, intravenous cannulation and use of vasoactive drugs which limits their use for a daily practice in clinical settings (La Rovere et al., 2008).

In this scenario, the sequence method emerges among the noninvasive alternatives to evaluate baroreflex in humans. Nevertheless, the concerns raised in this experimental study in rats should also be valuable for human subjects. Considering the average HR and respiratory frequency in humans, SAP ramps will also be limited to 3 to 4 cardiac beats. Therefore, similarly to what happens in rats, the sequence method in humans is also limited to assess spontaneous baroreflex function only at respiratory (fast) oscillations of SAP.

Therefore, the approaches suggested here to exclude the influences of respiration in spontaneous BRS in rats should also be applicable when the sequence method is used in signals from human beings.

In summary, we have confirmed the hypothesis that natural high-frequency components (in particular the respiration) of SAP and PI variability restricts the capability of the sequence method so that the slow components of the baroreflex are disregarded in the original method. To overcome this limitation, we proposed filtering out the high-frequency oscillations from SAP and PI series and use the sequence method with both original and LP filtered series, so that both slow and fast baroreflex function can be estimated. Nevertheless, different approaches could be considered to exclude the influence of respiration on SAP and PI (Topçu et al., 2018). Results point that our proposal seems to be a reasonable alternative to the classical approach. Further studies, with diversified datasets, are necessary to characterize the optimal parameters of the sequence method to be used with LP filtered time series.

## Data Availability

The raw data supporting the conclusions of this manuscript will be made available by the authors, without undue reservation, to any qualified researcher.

## Author Contributions

LS and RF conceived the study. CdS and DD collected the data. LS and DD performed the data analysis. LS, DD, HS, and RF analyzed the data. LS drafted the manuscript. LS, DD, CdS, HS, and RF revised and approved the final version of the manuscript.

## Conflict of Interest Statement

The authors declare that the research was conducted in the absence of any commercial or financial relationships that could be construed as a potential conflict of interest.

## Footnotes

1 www.danielpenteado.com
